# The Use of Androgen Deprivation Therapy for Prostate Cancer Lead to Similar Rate of Following Open Angle Glaucoma: A Population-Based Cohort Study

**DOI:** 10.3390/cancers15112915

**Published:** 2023-05-26

**Authors:** Po-Jen Yang, Chiao-Wen Lin, Chia-Yi Lee, Jing-Yang Huang, Ming-Ju Hsieh, Shun-Fa Yang

**Affiliations:** 1 School of Medicine, Chung Shan Medical University, Taichung 402, Taiwan; cshy1030@csh.org.tw; 2 Department of Family and Community Medicine, Chung Shan Medical University Hospital, Taichung 402, Taiwan; 3 Institute of Oral Sciences, Chung Shan Medical University, Taichung 402, Taiwan; cwlin@csmu.edu.tw; 4 Department of Dentistry, Chung Shan Medical University Hospital, Taichung 402, Taiwan; 5 Department of Ophthalmology, Nobel Eye Institute, Taipei 115, Taiwan; ao6u.3msn@hotmail.com; 6 Department of Ophthalmology, Jen-Ai Hospital Dali Branch, Taichung 412, Taiwan; 7 Institute of Medicine, Chung Shan Medical University, Taichung 402, Taiwan; cshe961@csh.org.tw; 8 Department of Medical Research, Chung Shan Medical University Hospital, Taichung 402, Taiwan; 9 Oral Cancer Research Center, Changhua Christian Hospital, Changhua 500, Taiwan; 10 Department of Post-Baccalaureate Medicine, College of Medicine, National Chung Hsing University, Taichung 402, Taiwan; 11 Graduate Institute of Biomedical Sciences, China Medical University, Taichung 404, Taiwan

**Keywords:** androgen deprivation therapy, open angle glaucoma, epidemiology, age, database

## Abstract

**Simple Summary:**

The purpose of the current study is to survey whether the application of androgen deprivation therapy (ADT) is associated with the development of open angle glaucoma (OAG) using the National Health Insurance Research Database (NHIRD) of Taiwan. Our results showed that the prostate cancer with ADT group showed a significantly lower risk of OAG development compared to the control group, while the risk of OAG development in the prostate cancer without ADT group was similar compared to that in the control group. The application of ADT did not lead to higher incidence of subsequent OAG. Patients with known risk factor for OAG may receive the ADT management without increased risk of OAG development.

**Abstract:**

This study aimed to survey the effect of androgen deprivation therapy (ADT) on the development of open angle glaucoma (OAG) in prostate cancer using the data from national health insurance research database (NHIRD) of Taiwan. A retrospective cohort study was conducted and patients were regarded as prostate cancer with ADT according to related diagnostic, procedure and medication codes. Each prostate subject with ADT was matched to one patient with prostate cancer, but without ADT, and two participants without both prostate cancer and ADT; 1791, 1791 and 3582 patients were recruited in each group. The primary outcome was set as the OAG development according to related diagnostic codes. Cox proportional hazard regression was used to estimate the adjusted hazard ratio (aHR) and 95% confidence interval (CI) of ADT for the incidence of OAG. There were 145, 65 and 42 newly developed OAG cases in the control group, prostate cancer without ADT group and prostate cancer with ADT group. The prostate cancer with ADT group showed a significantly lower risk of OAG development compared to the control group (aHR: 0.689, 95% CI: 0.489–0.972, *p* = 0.0341), and the risk of OAG development in the prostate cancer without ADT group was similar compared to that in the control group (aHR: 0.825, 95% CI: 0.613–1.111, *p* = 0.2052). In addition, ages older than 50 years old would lead to higher incidence of OAG development, respectively. In conclusion, the use of ADT will lead to a similar or lower rate of OAG development.

## 1. Introduction

Prostate cancer is a major malignancy in the male population [1], with more than 160,000 new patients diagnosed annually in the United State [2]. For treatment, androgen deprivation therapy (ADT) has been applied for decades which can reduce the function of prostate and suppress the progression of prostate cancer [1,3,4]. The treatment options of prostate cancer include the LHRH agonists, antiandrogens, estrogens and orchiectomy [5]. With the use of ADT, the median survival duration for prostate cancer was approximately 14 years [6], and the early ADT showed benefit for prostate cancer with nodal metastases [7].

There are several complications after ADT arrangement [8]. Cardiovascular diseases are the major concern in ADT management [8,9]. In previous studies [9], the patients receiving ADT were associated with higher rate of ischemic stroke and coronary arterial diseases [8]. Moreover, the incidence of sudden cardiac death was significantly higher in those who received ADT [10]. In addition, ADT is correlated to the development of deep vein thrombosis [11]. Other complications of ADT include the cognitive decline, depression, anemia, osteoporosis and diabetes [12,13,14].

The association between ADT and ocular disorders has seldom been reported. Glaucoma is an optic neuropathy which features high intraocular pressure (IOP) in most cases [15]. In the subtypes of glaucoma, open angle glaucoma (OAG) accounts for more than 80 percent of glaucoma cases and is correlated to certain systemic diseases including hypertension and diabetes mellitus (DM) [15,16]. Moreover, the correlation between OAG and ischemic stroke or coronary heart disease had been demonstrated in previous researches [17,18]. ADT can lead to a higher rate of cardiovascular disorders and related mortality [19], and a previous study showed that the incidence of OAG decreased in prostate cancer patients who received ADT therapy [20]. Accordingly, ADT may have the chance to alter the rate of OAG; however, further research is needed to confirm this.

The purpose of the current study is to survey whether the application of ADT would alter the incidence of OAG using the National Health Insurance Research Database (NHIRD) of Taiwan. The other potential predisposing factors for the OAG development were also included in the analysis model.

## 2. Materials and Methods

### 2.1. Data Source

This retrospective cohort study adhered to the Declaration of Helsinki, in 1964 following amendment, and was also approved by both the Institutional Review Board of Chung Shan Medical University (Project identification code: CS1-20108) and the National Health Insurance Administration. The need for informed consent from participants was waived by the same two institutions. The NHIRD of Taiwan contains claimed data of health insurance for nearly the whole Taiwanese population, which means about 23 million people. The interval of NHIRD was from 1 January 2000 to 31 December 2018. The available data from the NHIRD include the International Classification of Diseases, Ninth Revision (ICD-9) diagnostic code, International Classification of Diseases, Tenth Revision (ICD-10) diagnostic codes, demographic data, examination code, procedure code and the ATC codes for medications. We used the longitudinal health insurance database (LHID), 2005 version, in the current study, which is a sub-database from the NHIRD. In LHID 2005, about 2 million subjects were randomly selected from the NHIRD in the year 2005, and the data of these patients can traced from 1 January 2000 to 31 December 2018.

### 2.2. Patient Selection

**Figure 1:**
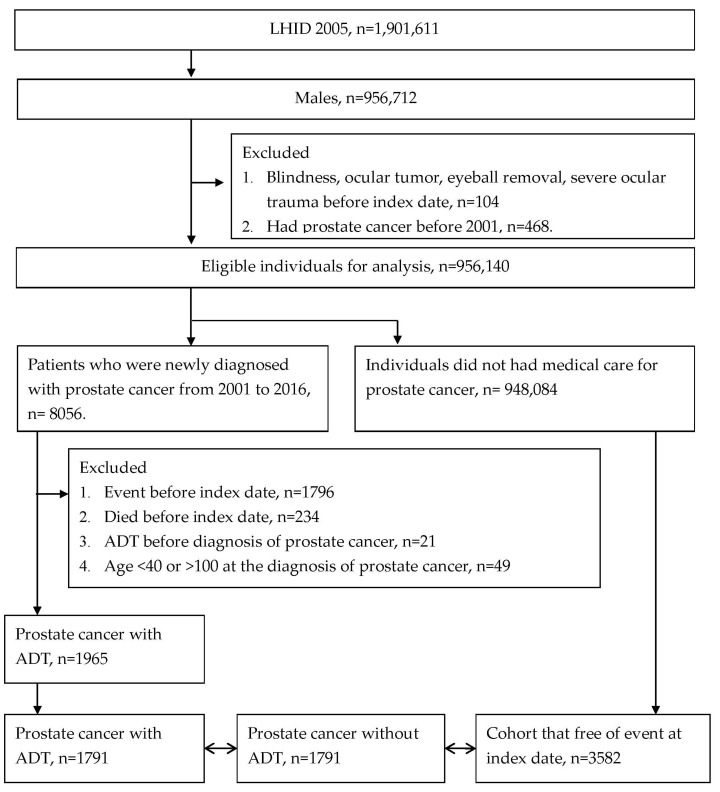
Flowchart of patient selection.

Men were regarded as prostate cancer with ADT if: (1) ICD-9/ICD-10 diagnostic codes indicated prostate cancer, (2) received LHRH agonists, antiandrogens, aromatase inhibitors, estrogens or bilateral orchiectomy based on associated procedure or ATC codes and (3) were in the age range from 40 to 100 years old. Additionally, the exclusion criteria of this study included blindness, ocular tumor, eyeball removal surgery, severe ocular trauma, outcome development or die before index date, ADT before prostate cancer diagnosis and prostate cancer diagnosis before 2001. Then, we used propensity-score matching (PSM) to match each patient in the ADT group to one prostate cancer patient who without ADT and two individuals without prostate cancer (the control group). The PSM was conducted according to age, year of index date and economic status. One subject with prostate cancer and ADT cannot be fully matched would be excluded from the current study. After all the processes, 1791, 1791 and 3582 patients were selected as the prostate cancer with ADT group, prostate cancer without ADT group and the control groups, respectively (Figure 1). The index date was defined as six months after the starting of ADT in prostate cancer participants.

### 2.3. Main Outcome Measurement

The primary outcome in our study is the development of OAG, which was regarded as: (1) the diagnosis of high-tension glaucoma and normal-tension glaucoma based on the associated ICD-9 or ICD-10 diagnostic codes, (2) the performance of fundus exam, optical coherence tomography or visual field exam before the diagnosis of OAG and (3) the OAG was diagnosed by an ophthalmologist. Because we want to evaluate the potential relationship between ADT and following OAG, only OAG developed after the index date was regarded as the achievement of the main outcome in the current study.

### 2.4. Demographic and Co-Morbidity Variables

To allow the overall status of the study population to be more homogenous, the effects of the following covariates were included in the analysis model: years of index date, age, urbanization, marital status, hypertension, DM, coronary arterial disease (CAD), acute myocardial infarction (AMI), hyperlipidemia, cerebrovascular disease and dementia. The existence of each co-morbidity was according to related ICD-9 or ICD-10 diagnostic codes. To be more specific, CAD referred to individuals diagnosed with chronic ischemic heart disease. All the participants were followed longitudinally from the index date till the date of OAG diagnosis, the date that withdrawal from the National Health Insurance program, or the end of NHIRD/LHID program, which means 31 December 2018.

### 2.5. Statistical Analysis

The SAS version 9.4 (SAS Institute Inc., Cary, NC, USA) was applied for the statistical analyses in the current study. After the PSM procedure, we used descriptive analysis to reveal the baseline characteristics of all the three groups. The Poisson regression was used to produce the incidence rate and corresponding 95% confidence interval (CI) of OAG among the three groups. Then, the Cox proportional hazard regression was utilized to demonstrate the crude and adjusted hazard ratio (aHR) of OAG among the three different groups which included the effects of demographic data and systemic diseases in the multivariable analysis. Then, the Cox proportional hazard regression was applied again to estimate the effect of each confounder on the development of OAG which presented with aHR and 95% CI. In addition, we drew Kaplan–Meier curves to illustrate the cumulative probability of OAG among the prostate cancer with ADT group, the prostate cancer without ADT group and the control group, and performed the log-rank test to calculate whether significant difference exist among the three groups. The statistical significance level was set at *p* < 0.05 in this study.

## 3. Results

The basic characteristics among the three groups are demonstrated in Table 1. The distribution of the age at index date, the year of index date, the urbanization state and the marital status were all similar among the three groups due to the PSM procedure (all *p* > 0.05). In addition, the ratios of systemic diseases were also similar among the three groups (all *p* > 0.05). About the usage of ADT, the antiandrogens therapy was applied in 67.67% of participants in the prostate cancer with ADT group, which is the most commonly applied ADT (Table 1). On the other hand, there were 663 participants who withdraw from ADT during the study period (Table 1).

**Figure 2:**
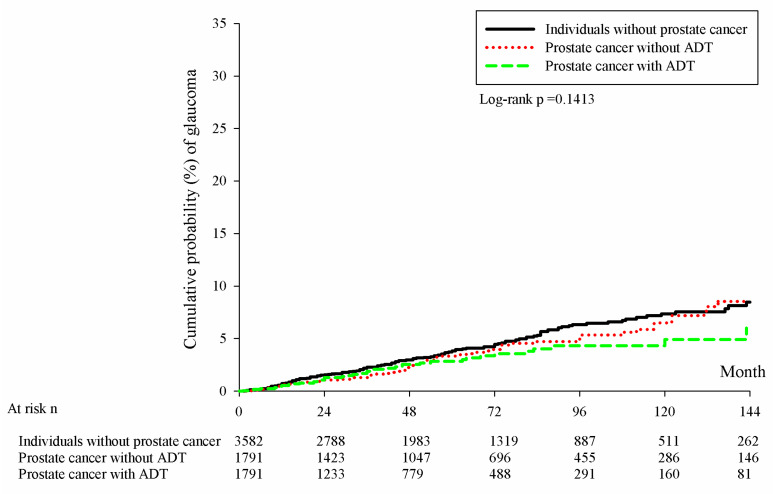
Kaplan–Meier curves with cumulative probability of open angle glaucoma among the three groups.

During the whole follow-up period, there were 145, 65 and 42 newly developed OAG cases in the control group, prostate cancer without ADT group and prostate cancer with ADT group, respectively (Table 2). According to the multivariable analysis, the prostate cancer with ADT group showed a significantly lower risk of OAG compared to the control group (aHR: 0.689, 95% CI: 0.489–0.972, *p* = 0.0341), while the risk of OAG in the prostate cancer without ADT group was similar compared to that in the control group (aHR: 0.825, 95% CI: 0.613–1.111, *p* = 0.2052). On the other hand, the Kaplan–Meier curves revealed a numerically lower cumulative probability of OAG in prostate cancer with ADT group (*p* = 0.1413) (Figure 2). Concerning the prominent predisposing factors other than ADT, the index date located between 2007 and 2011 would contribute to lower risk of OAG occurrence (*p* = 0.0287), while ages older than 50 years old would lead to higher incidence of OAG development, respectively (all *p* < 0.05) (Table 3).

## 4. Discussion

In the current study, the patient with prostate cancer and ADT revealed a lower incidence of OAG development compared to those with prostate cancer but without ADT and those without prostate cancer. Moreover, the incidence of OAG in the prostate cancer with ADT group was significantly lower than that in the control group. In addition, the patients older than 50 years old were associated with higher incidence of OAG development.

Several mechanisms are associated with the development of OAG [21]. The high IOP is the most important predisposing factor for OAG development [22], and the treatment program of OAG often consisted of IOP lower medications and surgeries [21]. However, some OAG, like the normal tension glaucoma, does not show an elevated IOP but the glaucomatous optic neuropathy including the retinal fiber thinning and visual field defect still occur [23]. In addition, some patients with raised IOP do not demonstrate glaucomatous optic neuropathy which is called ocular hypertension [24]. In these individuals who do not show the classic correlation of IOP and glaucomatous optic neuropathy, other pathophysiology including the vascular disorders have been purposed [25]. In the previous literature, the blood flow of optic nerve head would decrease in patients with glaucoma [26], and the superficial vessel density of both macular region and optic nerve head region were decreased in patients with OAG [27], which the loss of deep vessel density is more prominent in those with normal tension glaucoma [28]. In addition to the ocular vascular structure, systemic vascular disorders including hypertension, DM, CAD and cerebrovascular infarction had also been purposed as a risk factor for OAG development [15,16,17,18], and the existence of normal tension glaucoma is also related to subsequent ischemic stroke in a 10-year follow-up study [29]. In regard to ADT, the major complications are the metabolic syndromes, cardiovascular disease and deep vein thrombosis according to previous researches [30,31,32,33], in which the AMI had been reported to develop with a two-fold risk in patients who received ADT [34]. Since ADT is related to the development of vascular diseases which are related to OAG development, the use of ADT may showed some protective effect on the rate of following glaucoma which need to be evaluated.

In the current study, the use of ADT in prostate cancer was not correlated to a higher incidence of OAG development compared to those without prostate cancer and those prostate cancer individuals without the application of ADT. In fact, the aHR of OAG in the prostate cancer with ADT group was significantly lower than the aHR in the control group after adjusting multiple predisposing factors of OAG. To our knowledge, few papers have demonstrated this finding before. Only a study conducted in the Korean population demonstrated a significantly lower incidence of OAG in prostate cancer patients who received ADT compared to the prostate cancer patients who did not deceive ADT [20]. The results between that study and our study were similar while the study interval in our study was about two-fold compared to the previous study and could declare the long-term effect of ADT on OAG decrement [20]. In addition, the high level of urbanization is a predicting factor for glaucoma development which we adjusted in our multivariable analysis [35]. Together with the previous study, our study implies the universal correlation between ADT application and lower incidence of OAG in different ethnicities. Although the utilization of ADT is correlated to the occurrence of CAD and venous thrombosis event [17,33], reports of peripheral neuropathy or nephropathy after the arrangement of ADT are rare. We speculate that ADT may impair the large vessel including the cerebral artery or vein, but the effect of ADT on small vessels is limited. Additionally, ADT would cause the decrement of testosterone and the expression of testosterone would decrease the ocular blood flow [36], which is a significant risk factor for OAG development [26]. Other than ocular blood flow, the high level of testosterone was correlated to higher IOP in previous researches [37,38], and the injectable testosterone can directly induce OAG episode [39]. In addition to the direct effect, testosterone can elevate the blood pressure, in which hypertension is a known risk factor for glaucoma development [15,40]. Consequently, ADT might be a protective factor for OAG development due to the decrement of testosterone expression; however, further research is needed to clarify this concept. On the other hand, the cumulative probability of OAG did not illustrate significant difference among the prostate cancer with ADT group, prostate cancer without ADT group and the control group in the current study. Furthermore, the cumulative probability of OAG in the prostate cancer with ADT group did not excess the other two groups throughout the study interval, which may further demonstrate the safety of ADT management for OAG whether the ADT was ceased or not. 

Regarding the other potential predisposing factors for the development of OAG, the age would lead to a significant risk of OAG development. In previous studies, old age is a prominent risk factor for the development of all types of glaucoma, including OAG and the angle closure glaucoma [15,41,42]. Consequently, it is reasonable that the advanced age is correlated to the higher incidence of OAG development in the current study. Interestingly, the aHR of OAG was numerically decreased in those aged older than 80 years old. The possible explanation for this might be that patients older than 80 years old become less able to visit ophthalmic outpatient departments regularly. In regard to other established risk factor of OAG including hypertension and DM [15,22], no significant correlation between them and OAG occurrence were observed. Since we use the Cox proportional hazard regression for analysis, which considers the effect of more than 10 covariates, the effect of each parameter may be diminished. On the other hand, the demographic data are grossly similar among the three groups, which may demonstrate the fair homogeneity among all of our participants.

In the epidemiological aspect, OAG is the leading cause of visual impairment especially in high-income regions [43], and the prevalence of OAG was about six times more common than the primary angle closure glaucoma. [16] Despite the advancement of anti-glaucomatous medications [44], it remains the most frequent cause of irreversible blindness throughout the world [45]. On the other hand, prostate cancer is the second most commonly diagnosed cancer in the male population worldwide [46], and the ADT management is the standard treatment for both recurrent and non-recurrent prostate cancers [1,6]. Accordingly, surveying whether ADT would contribute to a higher risk of OAG is needed, and the current study illustrates the safety of ADT considering the development of OAG, at least to some extent.

There are certain limitations in the current study. Firstly, the retrospective design and the claimed data nature reduce the homogeneity among the different groups despite the PSM method. Second, we did not analyze the effect of different ADT separately because a number of patients used more than one type of ADT, while the cardiovascular effects result from each ADT could be different [47]. In addition, the numbers of primary outcome were few and may influence the statistical analysis. Besides, the result of optical coherence tomography and visual field exam cannot be obtained in the database, and thus, the severity of OAG is unknown. Furthermore, the influence of steroid application, which is a significant risk factor for OAG [48], was not analyzed in the current study because almost all the patients used some type of steroid at certain time points, which would lead to statistical error in the analysis. Finally, we found that ADT was discontinued in 663 participants while the reason for the cessation, due to resistance or other reasons, cannot be accessed since the real medical records were not available in the NHIRD/LHID 2005.

## 5. Conclusions

In conclusion, the utilization of ADT in patients with prostate cancer will lead to a similar rate, if not a lower rate, of OAG development after adjusting several risk factors for OAG. Furthermore, the long-term application of ADT contributes to minimal influence on the occurrence of OAG. Consequently, the use of ADT might be safe in elderly populations even if they have the risk factors of OAG. Further large-scale study to investigate whether the use of ADT would influence the clinical course of pre-existing OAG is mandatory.

## Figures and Tables

**Table 1:** Baseline characteristics among the three groups.

Character	Control (*n* = 3582)	Prostate Cancer without ADT (*n* = 1791)	Prostate Cancer with ADT (*n* = 1791)	*p* Value
Year of index				0.5434
2001–2006	856 (23.90%)	461 (25.74%)	442 (24.68%)	
2007–2011	1119 (31.24%)	570 (31.83%)	574 (32.05%)	
2012–2017	1607 (44.86%)	760 (42.43%)	775 (43.27%)	
Age				0.9607
<50	19 (0.53%)	9 (0.50%)	10 (0.56%)	
50–59	220 (6.14%)	101 (5.64%)	107 (5.97%)	
60–69	922 (25.74%)	453 (25.29%)	463 (25.85%)	
70–79	1498 (41.82%)	778 (43.44%)	737 (41.15%)	
≧80	923 (25.77%)	450 (25.13%)	474 (26.47%)	
Urbanization				0.8220
Urban	2017 (56.31%)	995 (55.56%)	982 (54.83%)	
Sub-urban	1160 (32.38%)	580 (32.38%)	596 (33.28%)	
Rural	405 (11.31%)	216 (12.06%)	213 (11.89%)	
Marital status				0.9165
Unmarried	87 (2.43%)	49 (2.74%)	58 (3.24%)	
Married	3151 (87.97%)	1560 (87.10%)	1547 (86.38%)	
Divorced	112 (3.13%)	65 (3.63%)	70 (3.91%)	
Widowed	232 (6.48%)	117 (6.53%)	116 (6.48%)	
Co-morbidities				
Hypertension	1907 (53.24%)	951 (53.10%)	961 (53.66%)	0.9389
DM	637 (17.78%)	336 (18.76%)	360 (20.10%)	0.1182
CAD	567 (15.83%)	287 (16.02%)	316 (17.64%)	0.2185
AMI	17 (0.47%)	10 (0.56%)	13 (0.73%)	0.5072
Hyperlipidemia	616 (17.20%)	291 (16.25%)	326 (18.20%)	0.3010
Cerebrovascular disease	430 (12.00%)	227 (12.67%)	238 (13.29%)	0.3920
Dementia	91 (2.54%)	47 (2.62%)	56 (3.13%)	0.4446
ADT type				
LHRH Agonists			1108 (61.86%)	N/A
Antiandrogens			1212 (67.67%)	N/A
Estrogens			140 (7.82%)	N/A
Bilateral orchiectomy			202 (11.28%)	N/A
Withdraw from ADT			663 (37.02%)	N/A

ADT: androgen deprivation therapy, AMI: acute myocardial infarction, *n*: number, CAD: coronary arterial disease, DM: diabetes mellitus, N/A: not applicable.

**Table 2:** Incidence risk of study event among the three groups.

Event	Control	Prostate Cancer without ADT	Prostate Cancer with ADT
Follow-up person months	230,050	119,285	93,479
New case	145	65	42
Incidence rate ^#^ (95% CI)	6.30 (5.36–7.42)	5.45 (4.27–6.95)	4.49 (3.32–6.08)
Crude Relative risk (95% CI)	Reference	0.864 (0.645–1.158)	0.716 (0.507–1.009)
aHR (95% CI)	Reference	0.825 (0.613–1.111)	0.689 (0.489–0.972) *

^#^ Incidence rate, per 10,000 person months; ADT: androgen deprivation therapy, aHR: adjusted hazard ratio, CI: confidence interval; * denotes significant correlation to open angle glaucoma development.

**Table 3:** Adjusted hazard ratio of open angle glaucoma from each covariate.

Covariate	aHR	95% CI	*p* Value
Group			
Control	Reference		
Prostate cancer without ADT	0.825	0.613–1.111	0.2052
Prostate cancer with ADT	0.689	0.489–0.972	0.0341 *
Year of index			
2001–2006	Reference		
2007–2011	0.718	0.534–0.966	0.0287 *
2012–2017	0.712	0.492–1.031	0.0723
Age at index			
<50	-		
50–59	Reference		
60–69	3.077	1.329–7.124	0.0087 *
70–79	3.621	1.576–8.323	0.0024 *
≧80	2.677	1.111–6.448	0.0281 *
Urbanization			
Urban	Reference		
Sub-urban	0.938	0.688–1.281	0.6886
Rural	1.022	0.629–1.660	0.9312
Marital status			
Unmarried	1.129	0.486–2.621	0.7783
Married	Reference		
Divorced	1.200	0.610–2.361	0.5974
Widowed	0.966	0.555–1.681	0.9028
Co-morbidities			
Hypertension	1.072	0.817–1.406	0.6154
DM	1.341	0.975–1.846	0.0716
CAD	1.031	0.733–1.449	0.8623
AMI	-		
Hyperlipidemia	1.012	0.711–1.440	0.9487
Cerebrovascular disease	1.054	0.712–1.561	0.7913
Dementia	0.449	0.110–1.824	0.2625

ADT: androgen deprivation therapy, aHR: adjusted hazard ratio, AMI: acute myocardial infarction, CAD: coronary arterial disease, CI: confidence interval, DM: diabetes mellitus; * denotes significant correlation to open angle glaucoma development.

## Data Availability

Restrictions apply to the availability of these data. Data were obtained from National Health Insurance database and are available from the authors with the permission of National Health Insurance Administration of Taiwan.

## References

[1] Gamat M., McNeel D.G. (2017). Androgen deprivation and immunotherapy for the treatment of prostate cancer. Endocr. Relat. Cancer.

[2] Teo M.Y., Rathkopf D.E., Kantoff P. (2019). Treatment of Advanced Prostate Cancer. Annu. Rev. Med..

[3] Leal F., Figueiredo M.A., Sasse A.D. (2015). Optimal duration of androgen deprivation therapy following radiation therapy in intermediate- or high-risk nonmetastatic prostate cancer: A systematic review and metaanalysis. Int. Braz. J. Urol..

[4] Gourdin T. (2020). Recent progress in treating advanced prostate cancer. Curr. Opin. Oncol..

[5] Crawford E.D., Heidenreich A., Lawrentschuk N., Tombal B., Pompeo A.C.L., Mendoza-Valdes A., Miller K., Debruyne F.M.J., Klotz L. (2019). Androgen-targeted therapy in men with prostate cancer: Evolving practice and future considerations. Prostate Cancer Prostatic Dis..

[6] Harris W.P., Mostaghel E.A., Nelson P.S., Montgomery B. (2009). Androgen deprivation therapy: Progress in understanding mechanisms of resistance and optimizing androgen depletion. Nat. Clin. Pract. Urol..

[7] Messing E.M., Manola J., Yao J., Kiernan M., Crawford D., Wilding G., di’SantAgnese P.A., Trump D. (2006). Immediate versus deferred androgen deprivation treatment in patients with node-positive prostate cancer after radical prostatectomy and pelvic lymphadenectomy. Lancet Oncol..

[8] Gruca D., Bacher P., Tunn U. (2012). Safety and tolerability of intermittent androgen deprivation therapy: A literature review. Int. J. Urol..

[9] Melloni C., Roe M.T. (2020). Androgen deprivation therapy and cardiovascular disease. Urol. Oncol..

[10] Keating N.L., O’Malley A.J., Smith M.R. (2006). Diabetes and cardiovascular disease during androgen deprivation therapy for prostate cancer. J. Clin. Oncol..

[11] Klil-Drori A.J., Yin H., Tagalakis V., Aprikian A., Azoulay L. (2016). Androgen Deprivation Therapy for Prostate Cancer and the Risk of Venous Thromboembolism. Eur. Urol..

[12] Andela C.D., Matte R., Jazet I.M., Zonneveld W.C., Schoones J.W., Meinders A.E. (2021). Effect of androgen deprivation therapy on cognitive functioning in men with prostate cancer: A systematic review. Int. J. Urol..

[13] Izard J.P., Siemens D.R. (2020). Androgen Deprivation Therapy and Mental Health: Impact on Depression and Cognition. Eur. Urol. Focus.

[14] Chen Y.Z., Chiang P.K., Lin W.R., Chen M., Chow Y.C., Chiu A.W., Tsai W.K. (2020). The relationship between androgen deprivation therapy and depression symptoms in patients with prostate cancer. Aging Male.

[15] Jonas J.B., Aung T., Bourne R.R., Bron A.M., Ritch R., Panda-Jonas S. (2017). Glaucoma. Lancet.

[16] Tham Y.C., Li X., Wong T.Y., Quigley H.A., Aung T., Cheng C.Y. (2014). Global prevalence of glaucoma and projections of glaucoma burden through 2040: A systematic review and meta-analysis. Ophthalmology.

[17] Chen Y.Y., Hu H.Y., Chu D., Chen H.H., Chang C.K., Chou P. (2016). Patients with Primary Open-Angle Glaucoma May Develop Ischemic Heart Disease More Often than Those without Glaucoma: An 11-Year Population-Based Cohort Study. PLoS ONE.

[18] Rim T.H., Lee S.Y., Bae H.W., Kim S.S., Kim C.Y. (2018). Increased stroke risk among patients with open-angle glaucoma: A 10-year follow-up cohort study. Br. J. Ophthalmol..

[19] Rosenberg M.T. (2020). Cardiovascular risk with androgen deprivation therapy. Int. J. Clin. Pract..

[20] Ahn H.K., Lee H.S., Park J.Y., Kim D.K., Kim M., Hwang H.S., Kim J.W., Ha J.S., Cho K.S. (2021). Androgen deprivation therapy may reduce the risk of primary open-angle glaucoma in patients with prostate cancer: A nationwide population-based cohort study. Prostate Int..

[21] Weinreb R.N., Aung T., Medeiros F.A. (2014). The pathophysiology and treatment of glaucoma: A review. JAMA.

[22] Weinreb R.N., Khaw P.T. (2004). Primary open-angle glaucoma. Lancet.

[23] Leung D.Y.L., Tham C.C. (2022). Normal-tension glaucoma: Current concepts and approaches—A review. Clin. Exp. Ophthalmol..

[24] Gordon M.O., Kass M.A. (2018). What We Have Learned From the Ocular Hypertension Treatment Study. Am. J. Ophthalmol..

[25] Flammer J., Orgül S., Costa V.P., Orzalesi N., Krieglstein G.K., Serra L.M., Renard J.P., Stefánsson E. (2002). The impact of ocular blood flow in glaucoma. Prog. Retin. Eye Res..

[26] Shiga Y., Kunikata H., Aizawa N., Kiyota N., Maiya Y., Yokoyama Y., Omodaka K., Takahashi H., Yasui T., Kato K. (2016). Optic Nerve Head Blood Flow, as Measured by Laser Speckle Flowgraphy, Is Significantly Reduced in Preperimetric Glaucoma. Curr. Eye Res..

[27] Rao H.L., Pradhan Z.S., Suh M.H., Moghimi S., Mansouri K., Weinreb R.N. (2020). Optical Coherence Tomography Angiography in Glaucoma. J. Glaucoma.

[28] Lee C.Y., Liu C.H., Chen H.C., Sun C.C., Yao Y.P., Chao S.C. (2019). Correlation between Basal Macular Circulation and Following Glaucomatous Damage in Progressed High-Tension and Normal-Tension Glaucoma. Ophthalmic Res..

[29] Lee M.S., Kuo L.L., Tan E.C., Lee O.K. (2017). Is normal-tension glaucoma a risk factor for stroke?—A 10-year follow-up study. PLoS ONE.

[30] Hu J.R., Duncan M.S., Morgans A.K., Brown J.D., Meijers W.C., Freiberg M.S., Salem J.E., Beckman J.A., Moslehi J.J. (2020). Cardiovascular Effects of Androgen Deprivation Therapy in Prostate Cancer: Contemporary Meta-Analyses. Arterioscler. Thromb. Vasc. Biol..

[31] Dawson J.K., Dorff T.B., Todd Schroeder E., Lane C.J., Gross M.E., Dieli-Conwright C.M. (2018). Impact of resistance training on body composition and metabolic syndrome variables during androgen deprivation therapy for prostate cancer: A pilot randomized controlled trial. BMC Cancer.

[32] Harrington J.M., Schwenke D.C., Epstein D.R., Bailey D.E. (2014). Androgen-deprivation therapy and metabolic syndrome in men with prostate cancer. Oncol. Nurs. Forum.

[33] Guo Z., Huang Y., Gong L., Gan S., Chan F.L., Gu C., Xiang S., Wang S. (2018). Association of androgen deprivation therapy with thromboembolic events in patients with prostate cancer: A systematic review and meta-analysis. Prostate Cancer Prostatic Dis..

[34] Carneiro A., Sasse A.D., Wagner A.A., Peixoto G., Kataguiri A., Neto A.S., Bianco B.A., Chang P., Pompeo A.C., Tobias-Machado M. (2015). Cardiovascular events associated with androgen deprivation therapy in patients with prostate cancer: A systematic review and meta-analysis. World J. Urol..

[35] Chan E.W., Li X., Tham Y.C., Liao J., Wong T.Y., Aung T., Cheng C.Y. (2016). Glaucoma in Asia: Regional prevalence variations and future projections. Br. J. Ophthalmol..

[36] Schmidl D., Schmetterer L., Garhöfer G., Popa-Cherecheanu A. (2015). Gender differences in ocular blood flow. Curr. Eye Res..

[37] Lee J.S., Lee M.H., Kim J.H., Jo Y.J., Shin J.H., Park H.J. (2021). Cross Sectional Study among Intraocular Pressure, Mean Arterial Blood Pressure, and Serum Testosterone according to the Anthropometric Obesity Indices in Korean Men. World J. Mens Health.

[38] Alpogan O., Donmez E.E., Balık A., Vural F., Kaplan G. (2021). Effects of testosterone on intraocular pressure, thicknesses of retinal nerve fiber layer, ganglion cell complex, macula and on ocular blood flow in female-to-male transgender persons. Int. Ophthalmol..

[39] Dahshan D., Verma V., Goel M. (2022). Open-Angle Glaucoma and Ischemic Optic Neuropathy with Injectable Testosterone Use. Ophthalmol. Glaucoma.

[40] Qu M., Feng C., Wang X., Gu Y., Shang X., Zhou Y., Xiong C., Li H. (2021). Association of Serum Testosterone and Luteinizing Hormone with Blood Pressure and Risk of Cardiovascular Disease in Middle-Aged and Elderly Men. J. Am. Heart Assoc..

[41] Gao F., Wang J., Chen J., Wang X., Chen Y., Sun X. (2021). Etiologies and clinical characteristics of young patients with angle-closure glaucoma: A 15-year single-center retrospective study. Graefes Arch. Clin. Exp. Ophthalmol..

[42] Gupta D., Chen P.P. (2016). Glaucoma. Am. Fam. Physician.

[43] Bourne R.R., Taylor H.R., Flaxman S.R., Keeffe J., Leasher J., Naidoo K., Pesudovs K., White R.A., Wong T.Y., Resnikoff S. (2016). Number of People Blind or Visually Impaired by Glaucoma Worldwide and in World Regions 1990–2010: A Meta-Analysis. PLoS ONE.

[44] Mehran N.A., Sinha S., Razeghinejad R. (2020). New glaucoma medications: Latanoprostene bunod, netarsudil, and fixed combination netarsudil-latanoprost. Eye.

[45] Stevens G.A., White R.A., Flaxman S.R., Price H., Jonas J.B., Keeffe J., Leasher J., Naidoo K., Pesudovs K., Resnikoff S. (2013). Global prevalence of vision impairment and blindness: Magnitude and temporal trends, 1990–2010. Ophthalmology.

[46] Kimura T., Egawa S. (2018). Epidemiology of prostate cancer in Asian countries. Int. J. Urol. Off. J. Jpn. Urol. Assoc..

[47] Agarwal M., Canan T., Glover G., Thareja N., Akhondi A., Rosenberg J. (2019). Cardiovascular Effects of Androgen Deprivation Therapy in Prostate Cancer. Curr. Oncol. Rep..

[48] Razeghinejad M.R., Katz L.J. (2012). Steroid-induced iatrogenic glaucoma. Ophthalmic Res..

